# Cardiovascular risk evaluation of HIV-positive patients in a case-control study: comparison of the D:A:D and Framingham equations

**DOI:** 10.7448/IAS.17.4.19515

**Published:** 2014-11-02

**Authors:** Samuel Markowicz, Marc Delforge, Coca Necsoi, Stéphane De Wit

**Affiliations:** Infectious Diseases, CHU Saint-Pierre, Brussels, Belgium

## Abstract

**Introduction:**

Patients with HIV infection are at increased risk of developing cardiovascular disease (CVD) due to complex interactions between traditional CVD risk factors, antiretroviral therapy (ART) and HIV infection itself [1]. Prevention of CVD is essential as it remains the most common serious non-AIDS event and contributes significantly to all-cause mortality. A cardiovascular risk-assessment model tailored to HIV population is thus essential.

**Materials and Methods:**

We conducted a retrospective case-control study within the HIV cohort of the Saint-Pierre Hospital, Brussels. Cases (*n*=73) presented a first CVD (ischemic heart disease or stroke) between January 2002 and December 2012. Controls (*n*=142) were patients without any CVD and were matched for age, race, sex and follow-up duration. We used Wilcoxon test to identify predictors of cardiovascular risk among the data collected. We compared Framingham [2] and DAD (Data Collection on Adverse Events of anti-HIV drugs) [3] equations calculated in all patients at time of event, two, four and six years before. We then simulated the impact on the DAD scores if different therapeutic interventions had been introduced when patient cardiovascular risk at ten years exceeded 20%.

**Results:**

Comparison of cases and controls showed that C-reactive protein (CRP) >3 mg/L (*p*=0.008) and HIV viral load >50 copies/ml (*p*=0.007) at time of event, as well as slower increase in CD4 cell count (*p*=0.035), were significantly more frequent in cases. DAD and Framingham median scores in cases and controls are shown in Figure 1 and Table 1. Smoking cessation lowered the DAD score of cases at time of event from 21.6% to 18.3%, modification of ART (discontinuation of indinavir, lopinavir and abacavir) lowered it from 21.6% to 17%, while both interventions with control of blood pressure and cholesterol lowered it from 21.6% to 12.4%.

**Conclusions:**

Increased CRP levels, uncontrolled HIV viral load at time of event and slower immunologic response were found to be associated with increased CVD risk. DAD score in cases increased more and faster over time than the Framingham score and seems therefore to be more accurate in identifying HIV-positive patients at high risk of CVD. Different therapeutic interventions could have led to a significant reduction of the DAD score in these patients and should remain a priority in patient management.

**Figure 1:**
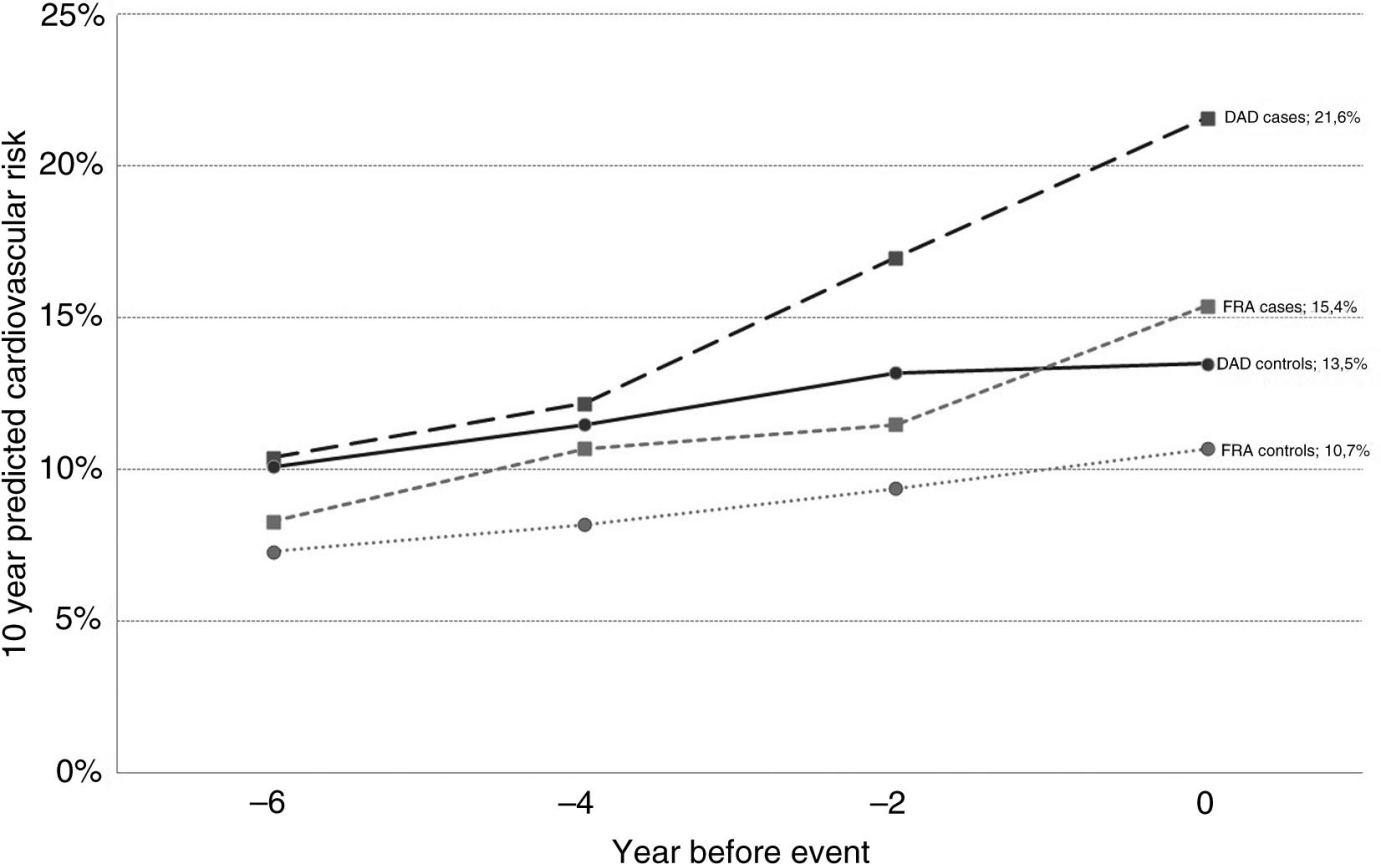
Ten-year predicted cardiovascular risk over time according to Framingham (FRA) and DAD equations in cases and controls.

**Table 1:** Framingham (FRA) and DAD scores at time of event, two, four and six years before, in cases and controls

Year before event	DAD cases (%)	DAD controls (%)	FRA cases (%)	FRA controls (%)
−6	10.4	10.1	8.3	7.3
−4	12.2	11.5	10.7	8.2
−2	17	13.2	11.5	9.4
0	21.6	13.5	15.4	10.7
